# A Revisit to the Pretargeting Concept—A Target Conversion

**DOI:** 10.3389/fphar.2018.01476

**Published:** 2018-12-17

**Authors:** Guozheng Liu

**Affiliations:** Department of Radiology, University of Massachusetts Medical School Worcester, MA, United States

**Keywords:** pretargeting, target conversion, tumor accumulation, quantitative correlation, semi-empirical model

## Abstract

Pretargeting is often used as a tumor targeting strategy that provides much higher tumor to non-tumor ratios than direct-targeting using radiolabeled antibody. Due to the multiple injections, pretargeting is investigated less than direct targeting, but the high T/NT ratios have rendered it more useful for therapy. While the progress in using this strategy for tumor therapy has been regularly reviewed in the literature, this review focuses on the nature and quantitative understanding of the pretargeting concept. By doing so, it is the goal of this review to accelerate pretargeting development and translation to the clinic and to prepare the researchers who are not familiar with the pretargeting concept but are interested in applying it. The quantitative understanding is presented in a way understandable to the average researchers in the areas of drug development and clinical translation who have the basic concept of calculus and general chemistry.

## Introduction

The pretargeting strategy (hereafter referred to as pretargeting) has been investigated for 30-odd years since its introduction to the field of radiotherapy (Reardan et al., 1985; Goodwin et al., 1986; Hnatowich et al., 1987). Because of its multiple injections, pretargeting is less investigated than the direct-targeting in which the effecting group is attached directly to the targeting antibody. Persistent efforts of several groups have advanced this strategy to clinic trials and the progress has been regularly updated (Goodwin, 1995; Goodwin and Meares, 1997; Barbet et al., 1999; Chang et al., 2002; Sharkey and Goldenberg, 2005, 2006, 2008, 2011; Goldenberg et al., 2006, 2008, 2012; Sharkey et al., 2007, 2010, 2012a,b; Goldenberg and Sharkey, 2010; Huang et al., 2011; Knight and Cornelissen, 2014; Kraeber-Bodéré et al., 2015; Larson et al., 2015; Bartholomä, 2018). This review focuses on understanding the pretargeting concept in a more quantitative way, in a hope to accelerate its development and translation to the clinic.

## The Nature of Pretargeting

**Figure 1:**
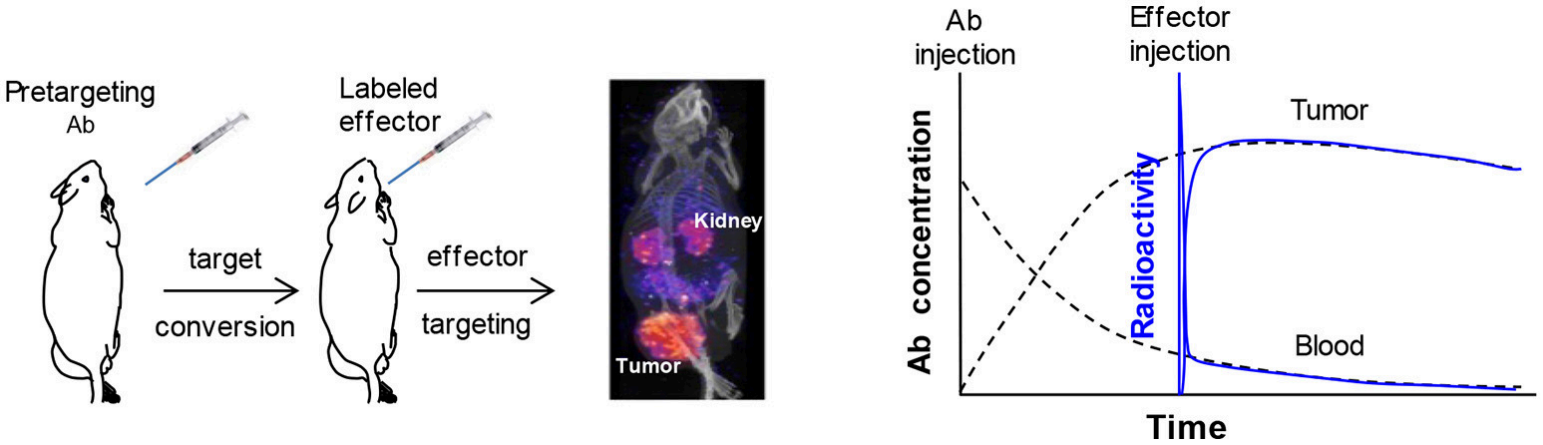
A typical pretargeting procedure and a schematic demonstration of the antibody concentration (in %ID/g) and the effector radioactivity (also in %ID/g) in the tumor and blood. Injection of the pretargeting antibody converts the antigen into a secondary target that binds the effector and injection of the effector allows the label to accumulate into tumor as illustrated in the pinhole SPECT image. In the schematic demonstration, the scales of antibody concentration and the effector radioactivity are adjusted to allow the curves for superimposition after the circulating free effector is cleared. It shows the concept of the *in vivo* labeling of the antibody and enables a better appreciation that the ratio of the tumor/blood area-under-curve for the effector radioactivity is much larger than that of the antibody.

Pretargeting prelocalizes an antitumor antibody into the tumor before injecting a small radiolabeled effector to recognize and specifically bind to it. The effector is excreted rapidly if not bound to the antibody. Pretargeting is widely viewed as a separation of tumor targeting of the antibody (pretargeting process) from the radioactivity administration to avoid the radiation exposure during the long process of antibody targeting (Goodwin, 1995; Goodwin and Meares, 1997). Figure 1 demonstrates the pretargeting process and schematically illustrates the tumor and blood curves of a labeled antibody in a direct-targeting strategy and a effector in a pretargeting process using that antibody. It helps to appreciate how pretargeting improves the tumor to blood ratio (and the ratios of the area-under-the radioactivity-curves, i.e., the therapeutic indexes) (Sung and van Osdol, 1995; Magnani et al., 1996; Pagel et al., 2003; Subbiah et al., 2003; Karacay et al., 2005; Sharkey et al., 2005). The effector curve (%ID/g) rapidly superimposes on that of the direct-targeting labeled antibody. It seems like the pretargeting antibody can be labeled *in vivo*, if assuming the labeled antibody and the pretargeting antibody behaves identically. However, it does not mean the percent tumor accumulation of the effector in a pretargeting setting is the same as that of the antibody in the direct-targeting setting. In fact, due to rapid clearance of the effector, its tumor accumulation is much lower than that of the antibody. The scales of the antibody concentration and the effector concentration (proportional to the radioactivity) are not the same and have been adjusted for the superimposition after the free effector is excreted.

We can also view pretargeting as a strategy to convert natural antigens into a secondary target specific for the small effector. This understanding helps to quantitatively comprehend how the tumor accumulation (%ID/g) of the effector depends on the antibody dosage, pretargeting interval (the time between the injections of the antibody and the effector), effector dosage, detection time (the time after radioactivity injection), and the properties of the effector, tumor, and tumor host. A natural question from this understanding is the necessity of the conversion. If a small targeting molecule is available that provides equally excellent target accumulation and comparable target to non-target (T/NT) ratios to that of the radiolabeled effector, the answer is no. Nevertheless, chances are such a small targeting molecule (or its target) may not be available. It is not easy to design a structure to provide both a high binding affinity to the tumor and a low normal tissue background simultaneously (Haberkorn et al., 2017; Kopka et al., 2017). Although most nuclear medicine imaging agents are small labeled molecules, few generate high target to non-tumor ratios and thus most are not satisfactory for tumor therapy (Herrmann et al., 2017; Bartholomä, 2018; Tsai and Wu, 2018). Also, a small targeting agent is specific for one target but an established pretargeting system can be applied to many antibodies that target different antigens.

We note that the target conversion comes at some expenses. In addition to the complexity of 2 injections, as a side effect the residual pretargeting antibody in the circulation binds the later-injected effector. In other words, pretargeting unfavorably creates an undesired low level of secondary targets in the circulation. Waiting for a period to allow the pretargeting antibody to be excreted from the circulation and optimizing the effector dosage can mitigate this side effect, but it is only a compromised solution. Use of a clearing agent as an additional injection would be more effective and may be a real solution. When the tumor accumulation is close to the maximum, injection of the clearing agent will bind and clear the residual circulating pretargeting antibody into liver. In the section of “3-Step pretargeting,” we will discuss this in more detail. If the effector is properly dosaged, the blood and normal tissue effector levels will be minimal while the tumor accumulation (%ID/g) will not be compromised by use of a clearing agent. As a result, much higher target to non-tumor (also abbreviated as T/NT) ratios can be provided to allow for a higher radioactivity dosage and more effective radiotherapy.

## Different Pretargeting Mechanisms

**Figure 2:**
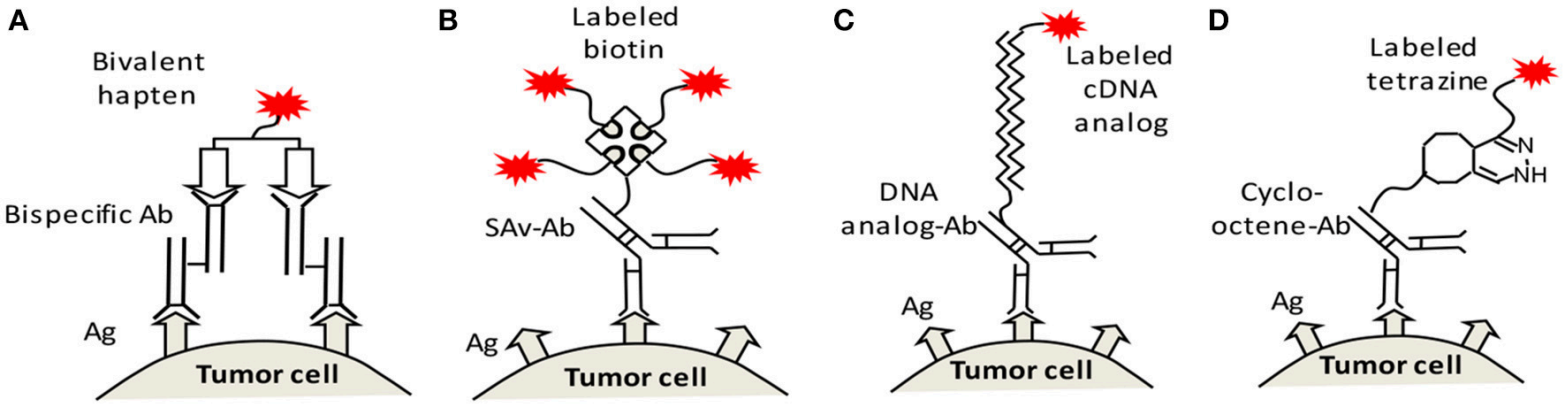
Four recognition mechanisms for pretargeting, namely **(A)** bispecific antibody/bivalent hapten, **(B)** streptavidin (SAv)/biotin, **(C)** DNA/cDNA analogs, and **(D)** *in vivo* clicking reaction (shown as an example is the reaction between cycloctene and tetrazine). Ag, antigen; Ab, antibody; SAv, streptavidin.

There are several mechanisms for pretargeting, i.e., several different recognition pairs of secondary target (on the antibody)/effector. At least four mechanisms have been employed, namely the bispecific antibody/hapten(Reardan et al., 1985), (strept)avidin/biotin(Hnatowich et al., 1987), DNA/cDNA analogs(Kuijpers et al., 1993), and *in vivo* click reaction(Agard et al., 2004). These mechanisms each have their own advantages, but their nature and function are the same. They convert the primary natural antigens to a secondary target for a small effector, to allow for a low normal tissue background and high T/NT ratios. Figure 2 illustrates the final structures of the linkages between the labels and antigens on the tumor cell surface. We note that the entire structure may internalize after binding to the tumor.

The bispecific antibody/hapten mechanism was first proposed (Reardan et al., 1985). It initially experienced a low tumor retention due to the low binding affinity of the effector to the secondary target. The effector binding was later improved by use of bivalent haptens (Barbet et al., 1999). Although it has not become a standard of care at this time, this mechanism is continually being tried in the clinic or investigated preclinically (Goldenberg et al., 2006, 2012; Schoffelen et al., 2013, 2014; Bodet-Milin et al., 2016; Cheal et al., 2016, 2017). For example, Dr Larson's group recently reported a therapeutic study using the DOTA-PRIT approach, in which a bispecific antitumor antibody engineered by Dr. Wittrup's lab was used to bind the ^177^Lu-DOTA effector (Cheal et al., 2017). The preclinical success with tumor cure in mice may have benefited from the excellent pharmacokinetics of the ^177^Lu-DOTA, the highly efficient clearing mechanism, the long half-life of the ^177^Lu, and the multiple therapeutic cycles.

The (strept)avidin/biotin mechanism was proposed almost as early as the bispecific antibody/hapten mechanism. In the beginning, it attracted more attention probably because of the high affinity of the recognition pair (Hnatowich et al., 1987; Green, 1990). Prior to streptavidin, avidin was tested. But as we recently demonstrated (Dou et al., 2014a), after attaching to the antibody and injecting to animal or human body it drags the antibody into liver too rapidly. Thus, the chance of pre-localization of the secondary avidin target in the tumor was severely compromised. The later-used streptavidin has another problem of immunogenicity (Knox et al., 2000). In addition, streptavidin-antibody internalizes more rapidly and thus reduces the number of binding sites for the labeled biotin effector (Casalini et al., 1997; Muzykantov et al., 1999). Nevertheless, there continue to be investigations testing its clinical utility to target hematological malignancies (Frost et al., 2015).

The DNA/cDNA mechanism was introduced in 1993 (Kuijpers et al., 1993), much later than the two above-mentioned mechanisms. The initial *in vitro* test was successful but natural DNAs are not stable *in vivo*. The *in vivo* success was largely due to the advent of stable synthetic DNA analoges. The first successes in solid tumor targeting were achieved by use of the phosphorodiamidate morpholino oligomers (MORFs) (Liu et al., 2002b), although peptide nucleic acids (PNAs) were also considered (Rusckowski et al., 1997). Both MORFs and PNAs do not enter cells by themselves, excrete rapidly into urine and have no significant immunogenic response or obvious toxicity (Crooke, 1997). Our choice of MORFs was only based on their better solubility, slightly lower liver accumulation, and easier availability to us (Mang'era et al., 2001). MORF/cMORF pretargeting has been very successful preclinically for both imaging and therapy (Liu et al., 2006, 2008a, 2010a,c, 2011, 2012; Liu, 2017). Very recently, a Sweden group has achieved similar successes using the PNAs (Westerlund et al., 2015, 2018; Honarvar et al., 2016; Altai et al., 2017).

The *in vivo* click reactions make the most recent recognition mechanism and currently are under active investigation (Agard et al., 2004; Chang et al., 2010; Rossin et al., 2010, 2013; Zeglis et al., 2013; Keinänen et al., 2017; Membreno et al., 2018; Meyer et al., 2018; Shi et al., 2018). The Staudinger reactions between phosphines and azides, the strain-promoted cycloadditions, and the inverse electron-demand Diels-Alder (IEDDA) reactions have been considered for recognizing biomolecule (Agard et al., 2004; Carroll et al., 2013; Knight and Cornelissen, 2014). The IEDDA reactions are compatible to the *in vivo* environment. In theory, The formation of a covalent link between the pretargeted antibody and the effector should favor the effector retention in tumor. However, it is not clear whether this is crucial because excellent tumor retention has been achieved using each of the above-mentioned mechanisms. When the affinity between the antibody and effector is at or higher than ~ 10^−14^ M, retention seems not an issue. Dr. Meares's group previously incorporated an *in situ* covalent bond formation into the bispecific antibody/hapten mechanism but did not observe significant improvement in terms of tumor accumulation (Butlin and Meares, 2006; Corneillie et al., 2006). There is a kinetic model reporting that a higher binding affinity after reaching a certain level (~10^−14^ M) will not significantly improve the tumor accumulation (Orcutt et al., 2011).

Another potential advantage of this new *in vivo* click pretargeting mechanism may be its “bioorthogonal” nature, i.e., natural absence *in vivo* and non-interference with the *in vivo* biological process (Nwe and Brechbiel, 2009). In fact, non-interference of the *in vivo* biochemical processes outside of the cells is a prerequisite for pretargeting. For example, the streptavidin/biotin recognition mechanism is less used because streptavidin triggers immune response, binds the endogenous biotin, and intensifies antibody internalization. However, there is no evidence for the bispecific antibody/hapten and DNA/cDNA mechanisms to disturb the *in vivo* biological processes. The haptens do not exist *in vivo* and do not target endogenous receptors. After internalization, the metal-chelate haptens residualize but that is an advantage for retention (Larson et al., 2015). Similar internalization would also happen for *in vivo* click pretargeting. The DNA/cDNA mechanism does not interfere with the biological processes outside of the cells either. There is no or minimal DNA or RNA sequence outside of the cells. The cDNA analog effector binds the DNA analog-antibody outside of the cells and on the cell surface by forming a duplex bond. In this case even if internalization happens (although little is known about their fate), nether the cDNA analog effector nor the DNA analog secondary target would bind the DNA or RNAs in the cells. Thus, both would not be interfering with the inside biochemical process. The DNA analog-antibody, if not picking up an effector and internalizing itself, does have a chance to bind the DNA or RNA inside of the cells. But if so, that would be a huge advantage. Researchers in other fields have long desired to send DNA analogs into cells for gene therapy (Olkkonen et al., 2018; Rangasamy et al., 2018).

## 3-Step Pretargeting—A Modification With On-Demand Rapid Clearance of the Pretargeting Antibody

As said above, one expense for converting the natural antigens to a secondary target is the residual circulating secondary target. Injection of a clearing agent when the tumor accumulation of the antibody is close to the maximum can effectively address this issue (Goodwin et al., 1984). This 3-step modified pretargeting was considered at the early stage of the pretargeting concept and had been used in the clinical trial of the SAv/biotin recognition mechanism (Knox et al., 2000). A clearing agent has so far not considered in the clinical trial of the bispecific antibody/hapten mechanism probably to reduce complexity (Goldenberg et al., 2012). However adding a clearing step is crucial for therapy (Liu, 2016), and researchers have considered incorporation of a clearance step into this mechanism (Mirallié et al., 2005; Cheal et al., 2016, 2017).

Discussion of different clearing agents is out of the scope of this review, but we want to emphasize that most clearance mechanisms utilize the secondary target of the effector. It can be easily understood that, while the clearing agent binds the secondary target on the circulating antibody, it would also consume some of the secondary target localized in tumor. This will reduce the number of the binding sites for the effector in the tumor. The net effect is the same as that of antibody internalization and use of a lower antibody dosage. Nevertheless, the effect is rarely experienced in the preclinical stage (Sharkey et al., 1997), because the binding site reduction can be disguised by the unchanged percent tumor accumulation of effector at low dosage. We have indicated that the percent tumor accumulation of the effector (%ID/g or %ID) will not change if a sufficient number of the binding sites remains in the tumor (Liu et al., 2010b), Also, it is fortunate that the circulating antibody is more accessible for the clearing agent than the antibody localized in tumor.

Preoccupation of the secondary target by the clearing agent can be avoided by using an additional secondary target. In other words, we and other have conjugate two secondary targets on to an antibody, one for the effector and the other for the clearing agent (Mirallié et al., 2005; Liu et al., 2010b). In this case, although the clearing agent may still bind the antibody in the tumor, it will not block the effector accumulation. We have obtained the proof of the concept using avidin as a model clearing agent. But avidin is not an ideal clearing agent because it has a too strong clearing power that leads to insufficient clearance of the antibody deep in the interstitial space (Dou et al., 2014a). The use of a milder clearing agent allows for an effective clearance (Cheal et al., 2014). In addition, there will be a stochastic effect in attaching two separate groups to an antibody, that generates a small fraction of undesired effector-binding antibody lacking the group for the clearing agent. We have developed a strategy to address this issue by combining the two groups prior to attaching to the antibody (Dou et al., 2015).

## Quantitative Relationships for the Tumor Accumulation and Normal Tissue Levels of the Effector

**Figure 3:**
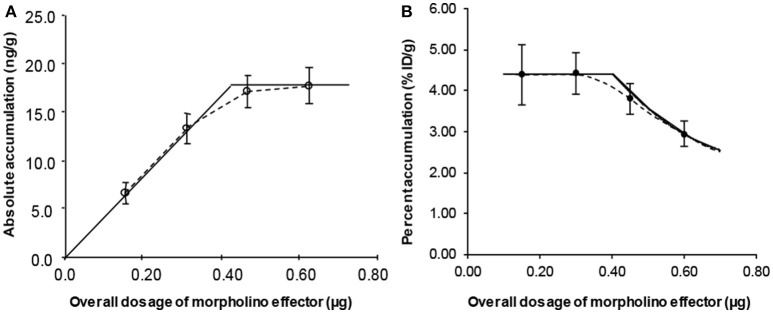
The percent **(A)** and absolute **(B)** tumor accumulations of a small cMORF against its dosage. The solid lines are obtained from our semi-empirical model rather than from regression fits.

Using a small labeled cDNA analog (^99m^Tc-cMORF) as the effector, we quantitatively investigated the influence of its dosage on its tumor accumulation in mice pretargeted 48 h earlier with a MORF-Ab (Liu et al., 2005). The percent accumulation of the injected effector per gram of tumor (%ID/g) is shown in Figure 3A. Figure 3B shows the absolute accumulation (ng/g) converted from the %ID/g. 4 injections of a small dosage are performed to observe the effect of fractionated injection (the exact contribution of each dosage increment). The 1st injection of the effector is given to all the 4 groups, 2nd injection to Groups 2–4, 3rd injection to Groups 3 and 4, and the 4th injection only to Group 4. All the mice were euthanized 3 h after the last injection. The injection intervals were set at 1 h apart because we have investigated the pharmacokinetics of cMORFs and known the targeting essentially completes in this period.

In Figure 3A, the overall %ID/g of ^99m^Tc-cMORF in tumor for the first 2 injections are the same, indicating occupation of some secondary targets in the tumor by the 1st injection does not impede the accumulation of the 2nd injection. This is the theoretical base for the tumor accumulation independence to the target number that we mentioned in the section of “3-Step pretargeting.” The overall %ID/g for 3 and 4 injections decline. After converting the %ID/g into the absolute accumulation (Figure 3B), it clearly indicates that after the 3rd injection the tumor can no longer take more cMORF effector, suggesting all the secondary targets have been occupied (saturated). Thus, the %ID/g before saturation is a constant and at the maximum. We referred to it as Maximum Percent Tumor Accumulation (*MPTA*). The saturation point is at the interception of the two straight lines in Figure 3B, the perpendicular line crossing the point of Group 4 and the straight line crossing the first two data points (Groups 1 and 2). The solid lines in Figure 3A are converted from the dotted lines in Figure 3B. Without this saturation theory, it is not possible to draw such a curved line. We later confirmed this saturation theory in another two studies (Liu et al., 2007a, 2010c). Dr Larson's group also observed similar phenomenon using the bispecific antibody/hapten mechanism, though obviously the lines would deviate from the experimental data a little, probably due to the two binging affinities of the bivalent effector (Cheal et al., 2016).

**Figure 4:**
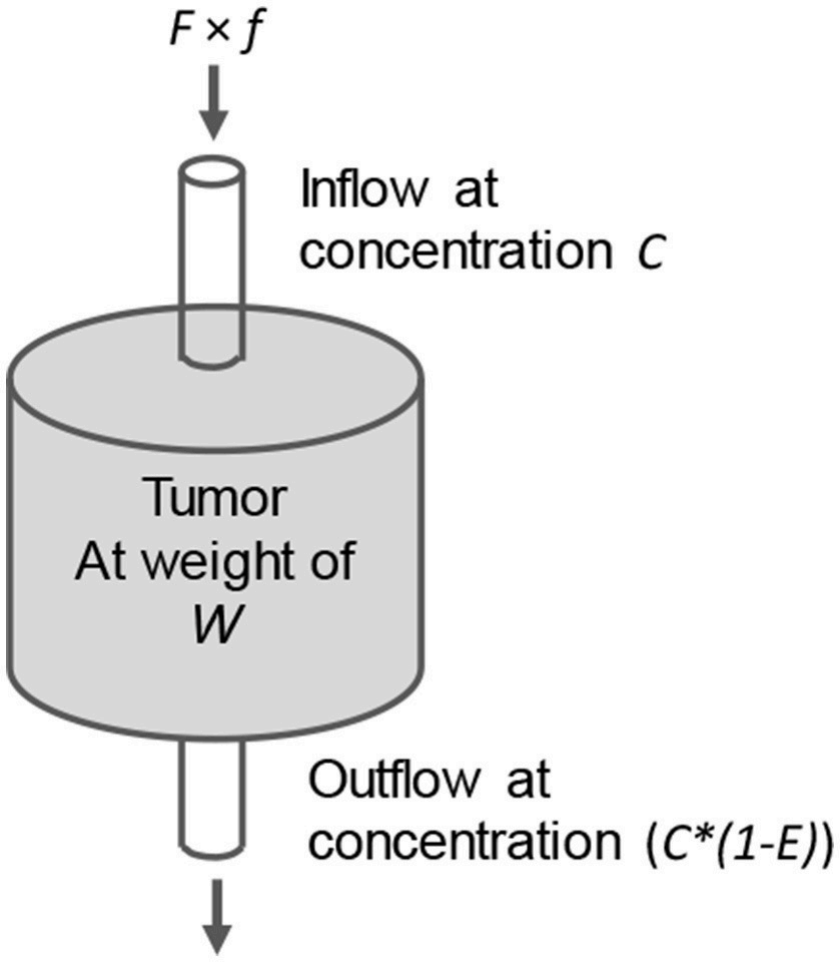
The “chemical reactor” model showing the inflow and outflow of a targeting molecule in the tumor (like a chemical reactor). *E* is the trapping efficiency of the targeting agent. Its accumulation *dQ (ng)* = *F* × *f* × *C (ng/g)* × *E* × *dt*.

We have established a semi-empirical tumor accumulation model to rationalize the above observations (Liu, 2013). Although there are more sophisticated tumor targeting models (Wittrup et al., 2012), this semi-empirical model works better for us. Unlike most compartment kinetic models (Carson, 1996; Wittrup et al., 2012), we do not need to fit previous experimental data for parameters required by the model. Instead, we measure them directly from experimental data. We refer to our model as a “chemical reactor” model (Figure 4). In other words, we consider the tumor like a “chemical reactor” and treat the agent accumulation based on the principle of mass conservation. Like most compartment models, this model is a lumped model. It does not differentiate the heterogeneous nature of a tumor but describes the tumor as a whole. It is just like “a chemical engineer forget to turn on the agitator” and, although the reactions inside the reactor is heterogeneous, the principle of mass conservation still apply. Lumped models are suitable for the studies that measure lumped properties including tumor weight, size, and accumulation of the targeting agent. In Figure 4, the blood flow (inflow) to the tumor is the product of the cardiac output *F* and its fraction to the tumor *f* . The tumor weight is represented by *W*, the tumor trapping efficiency of the targeting agent by *E*, and the area under the concentration curve by *AUC*_blood_ (input function). This model has been used to guide our optimization of pretargeting regimens (Liu et al., 2005, 2007b, 2009b; Liu and Hnatowich, 2008; Dou et al., 2014b).

When a tumor targeting agent is injected into the circulation, the blood flow carries the targeting molecules to the tumor. As shown in Figure 4, a fraction *E* is trapped there. Based on the principle of mass conservation, in an infinite small interval *dt* for a gram of tumor, the accumulation *dQ* of a targeting agent that binds the tumor with infinite affinity is:

(1) $$\begin{array}{r} dQ\;\left(ng/g\right)=F\left(mL/Min\right)\times f\times W^{-1}\left(g\right)\times E\times C\left(\frac{ng}{mL}\right)\times dt\;\left(Min\right) \end{array}$$

where *C* is the blood concentration of the targeting agent (ng/mL; assuming 1 mL of blood weighs a gram, the concentration can also be presented as ng/g). It tells a linear relationship of the accumulation rate to the blood concentration (C) just like the one-compartment model does: dQ = *k* × C × dt (Carson, 1996), but it further indicates *k* is related to *F, f, W*, and *E*.

Theoretically, starting from the bolus injection, the targeting continues for an indefinitely long period (∞) and the final accumulation is (the units will be omitted hereafter unless necessary):

(2) $$Q=\;\int_{t=0}^{t=\infty }F\times f\times W^{-1}\times E\times C\times dt$$

Dividing both sides of the equation by the injected dosage (in *ng*), we have the percent tumor accumulation (*PTA*) per gram of tumor:

(3) $$PTA\;\left(\%ID/g\right)=F\times f\times W^{-1}\int_{t=0}^{t=\infty }E\times C{\left(\%ID/g\right)}_{blood}\times dt$$

Although in theory tumor accumulation continues for an infinitely long time, it essentially completes when the time is sufficiently long, for examples 1 h is enough in mice for the pretargeting effectors and 2 d for the labeled IgG antibodies.

### Tumor Accumulation at Non-Saturating Dosages

In Figure 3, the first injection does not impede the accumulation of the second, indicating that before target saturation *E* for a small molecule with a very high affinity should be a constant and thus can be pulled out of the integration. Also shown in Figure 3A is that in this case the *PTA* is at the maximum (*MPTA*, the height of the perpendicular line):

(4) $$\begin{array}{l} MPTA\;\left(\%ID/g\;\right)=F\times f\times W^{-1}\times E\int_{t=0}^{t=\infty }C{\left(\%ID/g\right)}_{blood}\times dt\\ \;=F\times f\times W^{-1}\times E\;\times AUC_{blood}n \end{array}$$

We note although this *MPTA* expression (Equation 4) is derived from the cMORF tumor accumulation in a pretargeted setting, it should also apply to direct targeting, because we did not put any constraint as to whether the target is primary or secondary. Consistent to the experiences and as taken for granted when performing a biodistribution assay, the biodistribution of a labeled compound before saturation is the same regardless of its dosage, unlike in a block study or using a very large dosage at which the targets can be saturated.

Equation 4 is of predicting power. In pretargeted mice the effector binds the secondary target that is converted from an antigen, but the properties of the antibody specific for the antigen are not in the equation. We have validated that regardless of the antibody used, the *MPTA* is the same (Liu et al., 2008b). Furthermore, although increasing the dosage of the pretargeting antibody would increase the number of the secondary target sites, the number of the target sites is not expressed as a parameter in Equation 4. Thus, the *MPTA* will be at the same value as long as the effector dosage is below the saturation point (Liu et al., 2007a). For the same reason, increasing the number of the secondary targets by a so-called “amplification pretargeting” would neither improve the *MPTA*. This deduction rationalizes the previous failure in improving *MPTA* by use of the amplification mechanism (Kassis et al., 1996; He et al., 2004).

### Tumor Accumulation at Saturating Dosages

When the dosage of a targeting agent is above the saturation point, the conditions for MPTA will no longer exist. The tumor accumulation will then be dictated by the target number. If one effector binds one secondary target, the number of target sites should be equal to the number of effector molecules bound to the target, therefore

(5) $$\begin{array}{l} PTA_{agent}\;\left(\%ID/g\right)\\ \;=\frac{target\;number\;per\;gram\;of\;tumor}{Number\;of\;targeting\;molecules\;injected}\times 100\% \end{array}$$

The target number (moles of the secondary target for pretargeting) per gram of tumor should be the product of the moles of antibody injected (dosage of antibody *D*_antibody_ over its molecular weight *M*_*antibody*_), the tumor accumulation of antibody (*MPTA*_*antibody*_ in %ID/g), the groups of secondary target per antibody (*gpm*), and the fraction of the secondary targets that are not internalized and still able to bind the effector in the tumor (α). The number of targeting molecules (moles of the effector) injected is the effector dosage (*D*_*effector*_) over its molecular weight (*M*_*effecto*__r_). Thus, the percent tumor accumulation of the effector will be:

(6) $$\begin{array}{l} PTA_{effcetor}\left(\%ID/g\right)\\ \;=\frac{{M_{effector}\;\times \;D}_{antibody}\times \;gpm\;\times \;\alpha \times \;MPTA_{antibody}\;}{M_{Antibody}\;\times \;D_{effector}\;} \end{array}$$

Equation 6 is the mathematical expression of the declining curve in Figure 3A. It also links the two Y scales in Figure 1 (*PTA*_*effector*_ vs. *MPTA*_*antibody*_). Because antibody internalization continues over time, the percent tumor accumulation of the effector may decrease when a long pretargeting interval is used (Lollo et al., 1994; Santos et al., 1995; Gautherot et al., 1998; Liu et al., 2005). In other words, the saturation dosage of the effector at a shorter pretargeting interval is larger than that at a longer pretargeting interval. If the effector dosage is reduced to match the decreased number of accessible secondary targets, the effector *PTA* at a longer pretargeting interval would be back to its *MPTA* (Equation 4). However, we should bear in mind that tumor growth during the pretargeting interval may continues, that also contributes to the reduced target number per gram of tumor. The target number per gram of tumor in the equation is that at the time of effector injection.

The percent tumor accumulation (*PTA*) can be converted to absolute tumor accumulation (*ATA*) by multiplying it by the effector dosage *D*_*effector*_:

(7) $$\begin{array}{l} ATA_{effector}\;\left(ng/g\right)\\ \;=\frac{{M_{effector}\;\times \;D}_{antibody}\times \;gpm\;\times \;\alpha \times \;MPTA_{antibody}\;}{M_{Antibody}}\\ \;=constant\;\left(at\;Maximum\right) \end{array}$$

This explains Figure 3 in which, given an antibody dosage (*D*_*antibody*_), the absolute tumor accumulation of the effector (*ATA*) at saturation dosages is a constant and at its maximum (referred to as maximum absolute tumor accumulation, *MATA*). The declining *PTA* curve in Figure 3A can then be mathematically expressed in a simpler way by simplifying Equation 6 to:

(8) $$\begin{array}{r} {PTA\;}_{effcetor}\left(\%ID/g\right)=\frac{MATA_{effcetor}\;\left(ng/g\right)}{D_{effector}\;\left(ng\right)}\times 100\% \end{array}$$

Without this mathematical deduction, it is impossible to fit the limited data in Figure 3A with this a declining curve.

The above reasoning also applies to direct targeting. As said, in a blocking study with pre- or co-injection of the cold targeting agent, it is well agreed that a reduced tumor accumulation would suggest a specific binding. For a tumor targeting antibody, there are documented reports showing reduced tumor accumulations in human (Lamberts et al., 2015), but there are also many studies showing unreduced tumor accumulation even at a very high dosage. In this latter case, caution needs to be taken, because unreduced percent tumor accumulation may be due to the large number of the highly expressed antigens (Liu et al., 2005).

### Independence of MPTA Ratio to Tumor Size

**Figure 5:**
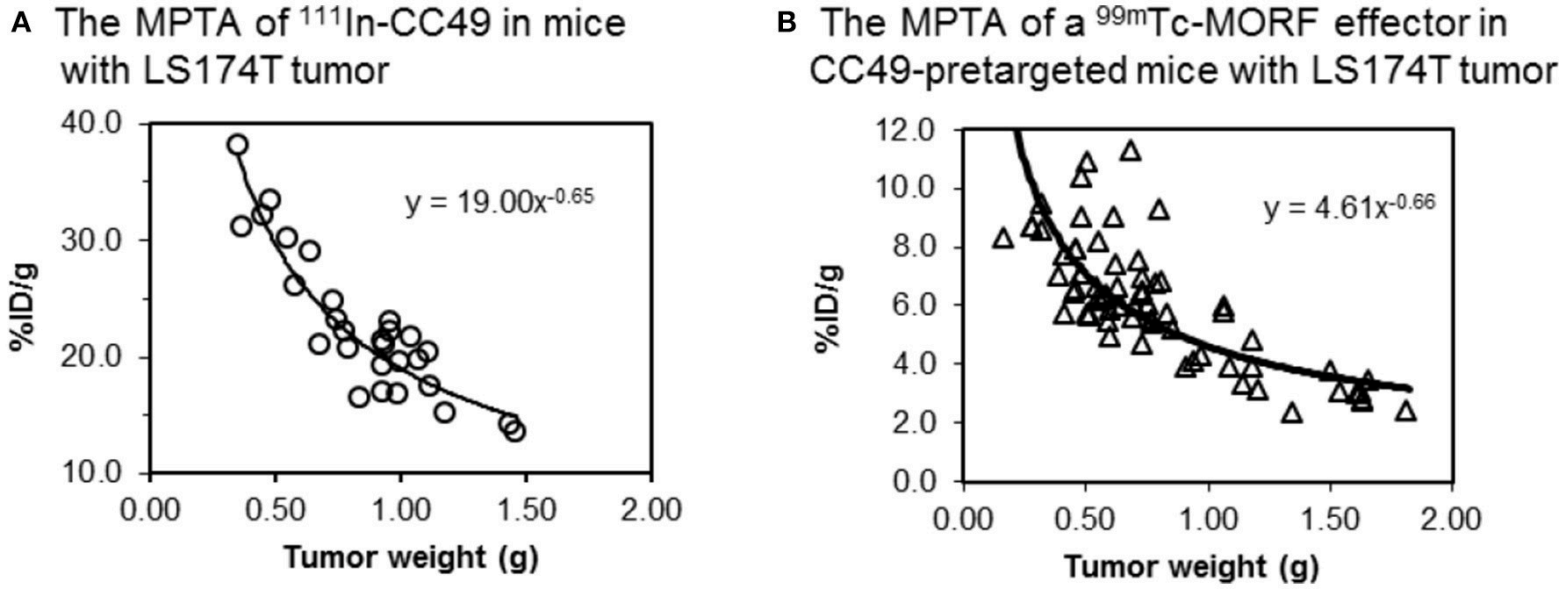
The contrast between **(A)** the MPTA of ^111^In labeled CC49 (~ 160 kDa) and **(B)** the MPTA of a small water-soluble ^99m^Tc-cMORF (~ 6 kDa) used in our pretargeting strategy. The cMORF clears essentially in 0.5–1 h, while it takes days for CC49. CC49 shows 4-fold higher tumor accumulation due to its longer circulation.

For a given tumor model at a given size in a given mouse strain, because *F, f, W, AUC*, and *E* are all fixed, the tumor accumulation (*MPTA*) is a constant. When tumor size varies, agreeing to that widely observed (Moshakis et al., 1981; Siegel et al., 1990; Sharkey et al., 2003b; Liu et al., 2008a), tumor accumulation (*MPTA*) may also vary. Our observation with the cMORF effector in a LS174T mouse tumor model pretargeted with a MORF-antibody is consistent to the reports in literature (Liu et al., 2009a). However, our surprising finding by further examination of our data is that while tumor accumulations of both the antibody and the labeled effector vary with the tumor size (Figure 5), their *MPTA* ratio is independent of the tumor size and remains constant (in this case, 4.12).

Based on Equation 4, this constant MPTA ratio leads to a constant *E* ratio because the *F, f*, and *W* are the same for the same tumor and the *AUC* is not a function of tumor size:

(9) $$\begin{array}{r} \frac{{MPTA}_{antibody}}{MPTA_{cMORF}}=\;\frac{E_{antibody}}{E_{cMORF}}\;\times \;\frac{AUC_{antibody}}{AUC_{cMORF}} \end{array}$$

Currently, tumor accumulation data for two different targeting molecules exactly in the same tumors are very limited. Thus, although very likely, whether the constant *MPTA* ratio of the two targeting agents is a common feature is yet to be fully validated.

### Normal Tissue Background in a Pretargeting Setting

The signal strength (%ID/g of the effector) in normal tissues reflects the total of the free effector and the effector bound to the residual circulating pretargeting antibody:

(10) $$\begin{array}{l} Total\;effector\;level=free\;effector\;level\;\\ \;+antibody-bound\;effector \end{array}$$

Without use of a clearing agent, the residual antibody level at the time of effector injection is appreciably high, though many times lower than that in the tumor. Due to its easier accessibility and lower level, the residual antibody is easily saturated. For all established pretargeting systems, the free effector levels in normal organs are very low after 1 h, while the level of circulating the antibody-bound effector may be considerable. Because of being distributed, it is not easy to be measured by imaging. We have noticed it can be calculated from the blood signal that can be measured by blood sampling (Liu et al., 2007a):

(11) $$\begin{array}{l} Total\;effector\;level=free\;effector\;level\;+blood\;level\;\\ \;\times \;organ/blood\;ratio \end{array}$$

As shown in Table 1, after correcting the free effector using equation 11, the organ to blood ratios of the antibody (or the antibody-bound effector) are constant, despite the effector levels (%ID/g) are very different from one study to another. This antibody equilibrium has been later confirmed by other researchers (Shah and Betts, 2013). Thus, except for the high free effector level in kidneys where it is excreted, the effector levels in other normal organs in a pretargeting setting can be calculated. When using a clearing agent, the principle applies, except the antibody-bound effector level is much lower. Equation 11 may be also very useful in clinical investigations because it is harder to measure the low normal tissue background by imaging.

**Table 1:** Percent levels (%ID/g) of ^99m^Tc-MORF effector in blood and several normal organs at 3 h post its IV injection to pretargeted mice and the organ to blood ratios of the antibody-bound effector^a^.

**Organs**	**Free^b^**	**Study 1^c^**	**Study 2^c^**	**Study 3^c^**	**Study 4^c^**	**Study 5^d^**	**Study 7^d^**	**Average**
Blood	0.04	5.44 ± 1.07	5.20 ± 0.48	3.34 ± 1.03	3.10 ± 0.46	1.27 ± 0.17	0.93 ± 0.08
Liver	0.27	1.90 ± 0.24	1.93 ± 0.18	1.33 ± 0.11	1.23 ± 0.15	0.60 ± 0.05	0.54 ± 0.03
Liver/blood		0.30	0.32	0.32	0.31	0.27	0.30	0.31
Spleen	0.17	1.09 ± 0.26	0.94 ± 0.22	0.70 ± 0.12	0.63 ± 0.08	0.33 ± 0.06	0.29 ± 0.05
Spleen/blood		0.17	0.15	0.16	0.15	0.13	0.13	0.15
Lung	0.12	2.04 ± 0.42	1.95 ± 0.19	1.37 ± 0.24	1.20 ± 0.30	0.74 ± 0.16	0.41 ± 0.07
Lung/blood		0.36	0.35	0.48	0.35	0.50	0.33	0.40
Heart	0.06	1.23 ± 0.18	1.05 ± 0.07	0.75 ± 0.07	0.71 ± 0.20	0.30 ± 0.07	0.22 ± 0.03
Heart/blood		0.22	0.19	0.21	0.21	0.20	0.18	0.20
Muscle	0.03	0.49 ± 0.08	0.45 ± 0.04	0.42 ± 0.09	0.36 ± 0.04	0.19 ± 0.02	0.13 ± 0.02
Muscle/blood		0.09	0.08	0.12	0.11	0.13	0.11	0.11

a *The organ/blood ratios are calculated based on Equation 11*.

b *The biodistribution of the free ^99m^Tc-MORF effector in non-pretargeted mice*.

c *(Liu et al., 2004)*;

d *(Liu et al., 2005)*.

## Optimization of a Pretargeting Regimen

Any change in antibody dosage, pretargeting interval, effector dosage, or detection time may lead to a different effector biodistribution. Adding a clearing agent would introduce more variables. Without a theoretical guide, a complete optimization of these variables would be difficult, especially in the clinic (Axworthy et al., 2000; Wu, 2001; Sharkey et al., 2003b; van de Watering et al., 2014). However, with the above quantitative relationships, the optimization becomes much easier (Liu and Hnatowich, 2008).

### Antibody Dosage

There is only a convenient but no optimal antibody dosage. To diminish the influence of any possible low antigen expression in normal tissues, the antibody dosage should not be very low, though an antibody dosage beyond saturation of tumor antigens is not recommended, because it will end up suboptimal T/NT ratios of the antibody and thus T/NT ratios of the effector. Over-dosaging seldom happen in human due to the large body weight. On the contrary, in a preclinical mouse study there is little chance for a too low dosage. Most likely any low but tangible antibody dosage would be usable in mice, but for a preclinical therapeutic study, the antibody dosage needs to be higher to provide enough secondary target sites to take a sufficiently high effector dosage for effective therapy.

### Pretargeting Interval and Dosage of the Clearing Agent

Without a clearing agent, a longer wait (pretargeting interval) provides a lower level of the residual circulating antibody and higher T/NT ratios of the “accessible” antibody. We say accessible because the internalized antibody is not visible to the effector. The T/NT ratios of the accessible antibody defines or equals to the achievable (optimal) T/NT ratios of the effector. Although a longer pretargeting interval provides higher T/NT ratios, it also renders more internalization and reduces the number of accessible secondary targets. Thus, the time for effector injection is dictated by the accessible T/NT ratios that are acceptable. Use of a clearing agent would obviate the long wait and its injection should be given when the antibody tumor accumulation is close to the maximum. The dosage of the clearing agent needs to be optimized to be just enough to clear the circulating antibody, especially when the clearing agent shares the same secondary target with the effector. More than needed would unnecessarily consume some valuable secondary targets in the tumor. Because the clearance is rapid, the time between injections of the clearing agent and the effector injection is short.

### Effector Dosage

Effector dosage needs to be optimized to match the number of the accessible secondary targets in tumor. At the saturation point, the T/NT effector ratios are optimal while still at the *MPTA*. As the effector level after corrected for the free effector by Equation 11 is that bound to the antibody, these optimal T/NT effector ratios should be the T/NT ratios of the accessible secondary targets or the accessible antibody. Below saturation, the T/NT effector ratios are suboptimal and are lower than the T/NT ratios of the accessible secondary targets. In this case, the residual secondary targets in normal tissues are saturated, but the secondary targets in tumor are not. Above the saturation, the extra effector getting into tumor will just pass by. In this case, although the T/NT effector ratios will still be optimal, the tumor accumulation (%ID/g) will be lower (not at the *MPTA*).

The saturation point can be measured pre-clinically using an effector dosage escalation (Liu et al., 2005). One important question is about the shiftability of effector dosage optimized on one tumor model to another. As discussed above, we have some experience on tumors of different size. The *MPTA* ratio of the pretargeting agent to the effector is not changing although both accumulations vary in size. If the internalization extent of the pretargeting antibody, i.e., the fraction of the secondary targets that are not internalized and still able to bind the effector in the tumor (α), is similar, the constant *MPTA* ratio at the saturation point (optimal dosage) can then be translated to an unchanged optimal dosage ratio of the pretargeting agent to the effector, based on Equation 6 at the saturation point in the form of:

(12) $$\begin{array}{r} \frac{MPTA_{effector}\;}{MPTA_{antibody}\;}=\frac{\;D_{antibody}\;}{D_{effector}\;}\times \frac{M_{effector}\;\times \;gpm\;\times \;\alpha \;}{M_{Antibody}\;} \end{array}$$

Thus, although tumors at different size may have a different pattern in vascularization and interstitial pressure (i.e., a pattern with different blood flow fraction to tumor *f* , tumor weight *W*, and tumor trapping efficiency *E*), the optimal dosage ratio between pretargeting antibody and effector should be the same. Better appreciating this reasoning can be achieved by considering a vascularization pattern that induces a less accumulation of the pretargeting antibody may probably proportionally reduce the delivery of the effector. Similar reasoning may apply to shifting a pretargeting system from one tumor model to another (a different cell line with the same antigen using the same antibody for pretargeting). However, at this time this reasoning has never been verified in different tumor models experimentally.

Optimization in human is more challenging because both tumors and tumor hosts (the patients) are different. Nevertheless, at least we expect the dosage ratio optimized from one subject may likely be extended to another, if the following logic holds to be true:

If patient variation could be viewed as tumor model variation, based on Equation 9, our observation (E^*^AUC)_antibody_/(E^*^AUC)_effector_ = constant should still be held and the following would be true:

(13) $$\begin{array}{r} {\left[\frac{MPTA_{antibody}}{MPTA_{effector}}\right]}_{Patient\;1}=\;{\left[\frac{MPTA_{antibody}}{MPTA_{effector}}\right]}_{Patient\;2} \end{array}$$

At the saturation point (optimal dosage ratio) and assuming the antibody internalization (α) in different patients is the same ($\frac{M_{effector}\;\times \;gpm\;\times \;\alpha \;}{M_{Antibody}\;}\;$will then be the same), combination of Equations 12 and 13 provides:

(14) $$\begin{array}{r} {\left[\frac{D_{effector}}{D_{antibody}}\right]}_{optimal\;for\;patient\;1}=\;{\left[\frac{D_{effector}}{D_{antibody}}\right]}_{optimal\;for\;patient\;2} \end{array}$$

Currently, the available data are not sufficient to confidently support extrapolation of (E^*^AUC)_antibody_/(E^*^AUC)_effector_ = *constant* among different patients. Very likely, the different pharmacokinetics in different patients may generate different *AUCs*. Fortunately, *AUC* is measurable and, if a difference is found, a correction may be made. Also, the assumption of the same extent of internalization needs to be validated. Thus, at this time, Equation 14 may be considered only as a first-order approximation. If the logic is later confirmed to be true, our simple model would be demonstrated with a predicting power once again.

### Detection Time

For tumor therapy, detection time is not a factor although biodistribution changes with it. Tumor pretargeting is not appealing for diagnosis, due to the multiple injections but it is meaningful for imaging-guided pretargeted immunotherapy. Imaging may be performed at multiple time points to measure the tumor radioactivity curve to estimate the radiation absorbed dose to tumor. In this case, the concept of an optimal imaging time is not applicable as well.

## Advantages of the Pretargeting Strategy and its Unaccomplished Mission

The most important advantage common to all the pretargeting mechanisms is that they can be adapted to different tumor antigens if a slow-internalizing antitumor agent is available. The nature of different pretargeting mechanisms is the same and the function of all the different recognition pairs is to convert the natural primary antigens to an artificial secondary target specific for a small effector. What differentiates different mechanisms is their ease for technological improvements. For examples, the immunogenicity of streptavidin has made its clinical application more difficult (Larson et al., 2015); the versatility in labeling an effector with both a diagnostic and a therapeutic nuclide may enable a system for broader theranostic utility (Sharkey et al., 2003b; Liu et al., 2009b); and an easier modulation of the effector structure may offer an opportunity to tailor its pharmacokinetics(Liu et al., 2002a,c; Meyer et al., 2017); Their application to the clinic is common in combining with other auxiliary measures for improved therapeutic effect. These measures may include fractionated or multi-cycle therapy (Cheal et al., 2016, 2017), better clearing agent (Cheal et al., 2014; Dou et al., 2015), improved specific radioactivity (Liu et al., 2006), and use of a different nuclide (Heskamp et al., 2017).

Another major advantage of pretargeting is its ability to address the normal tissue background of directly-labeled antibody. Natural clearance of the circulating antibody in combination with the rapid clearance of the effector enables the high T/NT ratios compared to that of the directly radiolabeled antibody (Goodwin, 1995). While the 2-step conventional pretargeting greatly improves the T/NT ratios, the 3-step pretargeting using a highly efficient clearing agent completely fulfills the promise for a very low normal tissue background. In addition to application to radioimmunotherapy, the high T/NT signal ratios are also be potentially useful for detection of sparsely-populated micro-organs such as islets in connection to diabetes (Liu et al., 2011, 2012). Medical physics cannot delineate the micrometer-sized pancreatic islets that account for only 1–2% of the pancreas.

Although not often mentioned in the literature, an additional advantage of pretargeting is the dramatic background reduction and thus-improved the T/NT ratios in some normal organs. The mechanism is the sequestration of the pretargeting antibody in these organs. The small effector does not “see” the pretargeting antibody extensively accumulated in the liver, and thus only slightly accumulates in this organ (Liu et al., 2003, 2012). Similar sequestration happens in the spleen and lung. Sequestration also happens in the kidneys with some smaller tumor-targeting proteins. Some directly radiolabeled proteins highly accumulate in kidneys due to renal reabsorption. Nevertheless, by attaching an effector-binding group to them and using them as the pretargeting agents, the kidney accumulation of the effector becomes lower (Westerlund et al., 2015, 2018; Honarvar et al., 2016; Altai et al., 2017). Although the effector excretes through this organ, it does not enter the compartment where the proteins reside such that the kidney radioactivity accumulation is lower.

The multiple-injections for pretargeting are apparently a disadvantage, but the high T/NT ratios are crucial for radiotherapy (Larson et al., 2015; Liu, 2016). Of course, if both an antibody and a small molecule are available for the same target, like the prostate membrane specific antigen (PMSA) (Smith-Jones et al., 2000, 2003; Sharkey, 2005; Haberkorn et al., 2017; Kopka et al., 2017), and the directly labeled small targeting molecule can achieve a similar success in terms of high T/NT ratios, it would be preferable to use that small molecule due to the single injection. However, in many cases antibody pretargeting and small targeting molecules are not comparable, because their primary targets are different. A small molecule targeting agent for one target is not likely to be shifted to another target. Developing a completely new small direct-targeting agent requires the structure to bind the target strongly and at the same time to clear from normal tissues rapidly. In contrast, applying an established pretargeting system to a different antigen is so much easier than developing a small targeting agent, just as Dr Sharkey said, “the direct route may not be the best way to home” (Sharkey, 2005).

The non-internalization requirement of the antibody is another constraint to pretargeting. Most antibodies internalize, including the so-called non-internalizing antibodies. It is fortunate in most cases a considerable percent of the pretargeting molecules remain on the cell surface at the time of effector injection. We have proved both theoretically and experimentally that the *MPTA*_effector_ will not be compromised by use of different antibodies and antibody internalization (Mirallié et al., 2005; Liu et al., 2007b, 2010c). Recently other researchers have also confirmed preclinically that internalizing antibodies can be used for pretargeting (Sharkey et al., 2012b; Houghton et al., 2016). We note this is not to say internalization is not a problem for pretargeting. When developed into the clinical stage where the pharmacokinetics of the antibody is slower than in mice, the internalization issue may deteriorate to an extent that it becomes impractical (Casalini et al., 1997).

An unaccomplished mission for pretargeting is to achieve a high tumor accumulation that the pioneers have long hoped by using a small effector (Goodwin, 1995). In the beginning, it was thought a rapid targeting may provide a tumor accumulation as high as that of the pretargeting antibody while generating a low normal tissue background (Goodwin, 1995). Gradually it becomes recognized that the targeting process of an effector is nothing different to that of the small molecule direct-targeting (Kopka et al., 2017). The rapid targeting can not compensate the rapid clearance (Liu et al., 2009b; Goldenberg et al., 2012). Currently, there is a paucity of investigations directly comparing the tumor accumulations of an antibody with that of a small effector in the same tumors as we did (Liu et al., 2005; Dou et al., 2014b). There are studies comparing the iodine-labeled antibodies with the metal-labeled effectors. Deiodination makes an underestimated the tumor signal of the antibody (Axworthy et al., 2000). Even so, the accumulation of the antibody is double or triple higher. Sometimes tumor accumulation of an effector can be very high, due to tumor model variation (van Schaijk et al., 2005a,b), but effector tumor accumulation is still several times lower than that of the antibody. It seems as if we need a targeting agent that is “both large and small” to have both the advantages of high tumor accumulation and low background. Recently, there was a report suggesting small molecules can achieve a tumor accumulation as high as that of the antibodies (Orcutt et al., 2011; Wittrup et al., 2012), but caution needs to be taken because the agent circulation and excretion data are limited to a given class of molecules. The experimental data continued to show the tumor accumulations of small hydrophilic molecules are 3- times lower than that of the IgG antibodies.

## Author Contributions

The author confirms being the sole contributor of this work and has approved it for publication.

### Conflict of Interest Statement

The author declares that the research was conducted in the absence of any commercial or financial relationships that could be construed as a potential conflict of interest.
